# The evolving landscape of first-line and subsequent therapies in *EGFR*-mutated NSCLC: efficacy, resistance, and tolerability

**DOI:** 10.37349/etat.2026.1002370

**Published:** 2026-04-27

**Authors:** Carminia Maria Della Corte, Caterina De Rosa, Faiz Ul Haq, Floriana Morgillo

**Affiliations:** N.N. Petrov Research Institute of Oncology, Russian Federation; Department of Precision Medicine, University of Campania “Luigi Vanvitelli”, 80131 Napoli, Italy

**Keywords:** *EGFR*-mutant NSCLC, acquired resistance, antibody-drug conjugates (ADCs), precision oncology

## Abstract

The treatment paradigm for advanced non-small cell lung cancer (NSCLC) harboring *EGFR* mutations is undergoing a significant transition. While third-generation tyrosine kinase inhibitors (TKIs) like osimertinib have long served as the frontline standard, the emergence of heterogeneous resistance mechanisms requires more robust therapeutic strategies. This review evaluates the clinical impact of the MARIPOSA trial, which demonstrated the superior efficacy of combining the bispecific antibody amivantamab with lazertinib. Beyond improving progression-free and overall survival, this dual-inhibition approach fundamentally alters the clonal evolution of the disease by suppressing common escape routes, such as *MET* amplifications and secondary *EGFR* mutations. Furthermore, we explore the diversifying landscape of second-line interventions, including the rise of antibody-drug conjugates (ADCs) like Sac-TMT and patritumab-deruxtecan, dual PD-1/VEGF inhibitors, and novel fourth-generation TKIs. By integrating preclinical insights on drug-tolerant persister cells with late-phase clinical data, this article outlines a future for *EGFR*-mutant NSCLC management defined by precision sequencing and the proactive mitigation of molecular resistance.

## Introduction

Over the last decade, the management of *EGFR*-positive non-small cell lung cancer (NSCLC) has been fundamentally redefined by the emergence of highly effective third-generation tyrosine kinase inhibitors (TKIs). Osimertinib, which received regulatory approval in 2015, marked a pivotal shift due to its ability to irreversibly target both primary sensitizing mutations and the *T790M* resistance variant. Beyond its molecular selectivity, its enhanced ability to penetrate the blood-brain barrier, and its improved safety profile, relative to its predecessors, established it as a cornerstone of therapy. While clinical data from the FLAURA and AURA3 trials solidified the role of third-generation agents like osimertinib, lazertinib, and aumolertinib as first-line standards, metastatic *EGFR*-mutated disease remains incurable. The inevitable development of drug resistance, often lacking clear biomarker guidance at the time of progression, highlights the urgent need for more robust frontline combination strategies.

## Mechanisms of resistance to third-generation TKIs and combination with chemotherapy in first line

Mechanisms through which tumors evade *EGFR* inhibition are typically divided into two categories: *EGFR*-on-target mutations and the activation of alternate signaling (bypass) pathways [1, 2]. On-target resistance to osimertinib frequently involves *EGFR* gene modifications that prevent the drug from binding effectively, most notably the *C797S* mutation, seen in roughly 7% of cases, alongside rarer substitutions like *L792H* or *G796R*. Additionally, *EGFR* amplification has been described as an acquired mechanism of *EGFR* TKI resistance [3, 4]. Alternatively, resistance can emerge independently of the *EGFR* receptor via bypass signaling. *MET* amplification is the most prevalent of these, occurring in 7% to 15% of patients. Less frequent drivers include genomic rearrangements involving *ALK*, *RET*, or *BRAF*, as well as *HER2* amplification and *RAS*-pathway alterations [5–7]. Furthermore, tumors may undergo histological transformation, such as switching to a small-cell lung cancer (SCLC) phenotype. This transformation, found in up to 15% of patients, carries a poor prognosis and is frequently driven by the concurrent loss of the *RB1* and *TP53* tumor suppressors [8, 9]. Although many of these alterations represent potentially therapeutic candidates, increasing tumor heterogeneity, the absence of biomarker-guided approved therapies, and the frequent lack of identifiable actionable genomic resistance mechanisms collectively pose major challenges for treatment in later lines of therapy. Based on the good safety profile of third-generation TKIs, first-line implementation with combination therapy strategies may circumvent the occurrence of resistance and prolong first-line outcomes.

The development of combined treatment for *EGFR*-mutant NSCLC has been influenced by several trials initially designed to enhance the effectiveness of 1st- and 2nd-generation *EGFR* TKIs. Initial attempts to combine anti-*EGFR* agents with chemotherapy did not provide consistent evidence of improved overall survival (OS). More recently, progress has focused on combining 3rd-generation *EGFR* inhibitors with antiangiogenic agents, chemotherapy, and bispecific antibodies to enhance efficacy by targeting multiple pathways. A strategy that has been employed is to combine *EGFR* TKIs with chemotherapy, thereby leveraging the targeted activity of targeting *EGFR* alongside chemotherapeutic agents’ broader cytotoxic effects. Given the potential benefits of chemotherapy and the advancements in *EGFR* TKI development, combining chemotherapy with third-generation *EGFR* inhibitors became a logical next step. The FLAURA2 study explored the integration of platinum-based chemotherapy with osimertinib in treatment-naive patients with *EGFR*-mutated NSCLC. This combination led to a meaningful extension of progression-free survival (PFS) compared to osimertinib monotherapy (25.5 vs. 16.7 months) [10]. Recent survival analyses confirmed this benefit, showing a median OS of 47.5 months for the combination versus 37.6 months for the single agent. After three years, 63% of patients receiving combination therapy were still alive, compared to 51% of those receiving monotherapy [11]. This approach can be readily implemented in clinical practice due to thoracic oncologists’ expertise in administering chemotherapy and the relatively low cost and wide availability of chemotherapeutic agents. However, the addition of chemotherapy to targeted therapy in oncogene-addicted tumors must be carefully evaluated and applied in real-world settings. Important considerations include advanced patient age, cardiovascular comorbidities, and treatment adherence. These cases may still benefit from a single-agent osimertinib-based approach. On the other side, some genomic features (such as *TP53* co-mutations) or clinical disease-related aspects (like high tumor burden or liver/brain mets) may encourage and support the combination strategies.

Interestingly, the spectrum of acquired resistance mechanisms remained largely consistent between the two treatment arms. Fewer patients in the osimertinib-plus-chemotherapy group exhibited detectable acquired genomic alterations compared with those receiving osimertinib alone, and no novel resistance mechanisms were identified. While the addition of chemotherapy may increase toxicity, it appears to delay resistance without fundamentally altering the molecular profile of the escape mechanisms, which remain dominated by *C797S* and *MET* alterations [12, 13]. Among patients who proceeded to second-line therapy, chemotherapy was the most frequently administered treatment in both arms. Platinum-based regimens were given to 81% of patients previously treated with osimertinib monotherapy and 32% of those from the combination arm. Notably, 54% of patients in the experimental arm and 40% in the control arm who discontinued study treatment did not receive subsequent therapy, highlighting the urgent need to maximize benefit in the first-line setting. Interestingly, the addition of chemotherapy may not only delay the emergence of resistance but could also potentially simplify the heterogeneity of osimertinib-induced drug resistance profile by eliminating or inhibiting specific sensitive subclones. Therefore, this combination may create more favorable conditions for subsequent treatment strategies upon progression.

## Resistance to bispecific antibody against *EGFR* and *MET* and combination with *EGFR* TKIs in first line

A second key approach to combinatorial therapies involves the simultaneous targeting of multiple relevant pathways and the incorporation of common mechanisms of acquired resistance. As amplification of the *MET* gene is the most common cause of secondary resistance, numerous studies have evaluated dual EGFR–MET inhibition to enhance outcomes, both after resistance emerges and as a proactive frontline strategy. In the frontline setting, the results of the MARIPOSA trial showed that combining amivantamab, an EGFR–MET bispecific antibody, and lazertinib, an *EGFR* TKI, led to an OS benefit, establishing this regimen as a new standard of care. The amivantamab–lazertinib combination exerts a multifaceted ‘dual inhibition’ simultaneously down-regulating two distinct receptors (EGFR and MET) while also providing vertical inhibition of the EGFR pathway itself by combining a bispecific antibody with a TKI.

The MARIPOSA trial, a multi-center, randomized, open-label phase III clinical study, examined the efficacy of frontline dual inhibition using the bispecific antibody amivantamab alongside the third-generation TKI lazertinib. This investigation compared the combination against the standard-of-care, osimertinib, in treatment-naïve individuals diagnosed with advanced *EGFR*-mutated NSCLC. Participants were assigned in a 2:2:1 ratio to one of three arms: amivantamab with lazertinib, osimertinib monotherapy, or lazertinib alone. To ensure balanced cohorts, stratification was based on race, *EGFR* mutation subtype, and the existence of brain metastases. While PFS served as the trial’s primary objective, researchers utilized regular brain magnetic resonance imaging (MRI) monitoring for every patient to track intracranial disease, regardless of whether central nervous system (CNS) involvement was present at the start of the study. In terms of efficacy, the MARIPOSA trial successfully achieved its primary goal: patients receiving the amivantamab–lazertinib combination experienced a median PFS of 23.7 months, compared to 16.6 months for those on osimertinib. While the ORRs were nearly identical between the two groups (86% vs. 85%), the combination therapy significantly extended the duration of those responses to 25.8 months, compared to 16.8 months with the single agent. This finding underscores the potential of proactive combination strategies to sustain clinical benefit longer. Furthermore, the experimental arm showed superior results in several exploratory areas, including time to subsequent treatment, time to drug discontinuation, and PFS2. Most notably, the final analysis confirmed a statistically significant improvement in OS. The mOS was not yet reached for the combination group, vs. 36.7 months for osimertinib. A 3.5-year OS reached 56% with amivantamab–lazertinib compared to 44% with the control. The combination is estimated to provide a median OS advantage exceeding 12 months over standard monotherapy [14, 15]. Despite the superior efficacy of these combinations, the transition from osimertinib monotherapy to more complex regimens faces challenges regarding tolerability and patient compliance. The MARIPOSA regimen is associated with higher rates of infusion-related reactions and dermatological toxicities, while the FLAURA2 regimen introduces a known profile of chemotherapy-related haematological side effects. In real-world practice, patient stratification and preference may be crucial. Osimertinib monotherapy remains a vital standard for elderly patients or those with significant comorbidities, whereas aggressive frontline combinations may be prioritized for patients with high-risk features, such as *TP53* co-mutations or high TMB.

These positive data represent a confirmation of the biological rationale to introduce an innovative combination in clinical practice to delay progression in NSCLC with *EGFR* mutations, with the possibility to alter the biological story of the disease, targeting *MET* from the beginning. However, translating these data into clinical practice may be slow, not only for the limited access worldwide to amivantamab, but also for the need to improve oncologists’ skills in managing and proposing to patients a new drug, facing some safety issues, especially due to infusion-related adverse events. Similarly to chemo-based combination, disease high-risk features like visceral metastatic sites and the number of metastases may support the choice of a new combination in a patient with good performance status.

Notably, crossover was not available in the study, and receipt of amivantamab was uncommon at subsequent therapy off study. Approximately 25% of patients in both groups did not receive second-line therapy. Following the onset of disease progression, platinum-containing chemotherapy served as the primary subsequent treatment modality for the majority of patients. Specifically, among those who transitioned to a new line of therapy, chemotherapy was administered to 56% of patients in the combination group and 67% of those in the osimertinib group. Interestingly, the utilization of subsequent *EGFR* TKIs was observed more frequently in patients who had initially received the amivantamab–lazertinib combination compared to those treated with osimertinib monotherapy (39% vs. 28%).

Regarding potential biomarkers of selection for amivantamab and lazertinib combination, some data are already available. In the MARIPOSA study, investigators employed the Guardant 360 CDx next-generation sequencing platform to evaluate circulating tumor DNA (ctDNA) from 858 participants. This analysis relied on paired blood samples obtained at baseline and at the end of treatment (EOT), defined as the point of disease progression, therapy discontinuation, or within 90 days following cessation. Matched genomic data from both time points were successfully collected for 53% of patients in the amivantamab plus lazertinib group and 56% of those in the osimertinib group. The results indicated that the frontline dual-targeted strategy significantly suppressed the emergence of standard resistance pathways compared to osimertinib monotherapy. Specifically, acquired *MET* amplifications were observed in only 4.4% of patients receiving the combination versus 13.6% in the osimertinib arm. Furthermore, secondary *EGFR* resistance mutations—including *C797S*, *L718X*, and *G724X*—were remarkably lower in the combination cohort at 0.9% compared to 7.9% in the control group. These outcomes underscore the dual mechanism of amivantamab in simultaneously inhibiting the *MET* and *EGFR* pathways. Beyond these primary drivers, the incidence of acquired *TP53* resistance mutations was numerically lower in the experimental arm (9.7% vs. 12.9%). Similarly, the concurrent loss of *TP53* and *RB1*, which often facilitates transformation into SCLC histology, occurred in only 0.9% of patients on the combination versus 2.9% on osimertinib. While no significant differences were noted in other markers like *HER2* amplification or *RAS*/*RAF* and phosphatidylinositol 3-kinase (PI3K) pathway alterations, patients treated with osimertinib developed a more heterogeneous and complex mutational landscape, often characterized by multiple concurrent alterations. In contrast, the amivantamab and lazertinib regimen fostered a more limited resistance profile, suggesting that proactive *MET* targeting can fundamentally alter the biological evolution of the disease [16]. It is important to note that while initial data from trials like MARIPOSA provide genomic insights via ctDNA, detailed and histologically verified resistance mechanisms in the classic mutation population treated with amivantamab plus lazertinib remain under active investigation. For example, current findings regarding *HER2*-driven drug-tolerant persister (DTP) cells are primarily derived from preclinical models or small patient cohorts and necessitate further validation in larger clinical studies to be considered established clinical biomarkers.

Little research has been conducted into the predictive biomarkers and resistance mechanisms of amivantamab. Few preclinical studies have explored the resistance mechanisms that may emerge during amivantamab and lazertinib treatment. In a specific preclinical investigation, researchers examined the drug-tolerant environment created by the amivantamab and lazertinib combination using a patient-derived xenograft (PDX) model of treatment-naive NSCLC with an *EGFR* exon 19 deletion. This experimental model was established by transplanting fresh tumor tissue from an untreated patient into athymic nude mice. Through the use of single-cell RNA sequencing, scientists pinpointed a unique population of DTP cells specifically marked by an increase in *HER2* expression. Consistently, *HER2* immunohistochemistry of PDX tumors demonstrated that amivantamab–lazertinib therapy induced *HER2* overexpression. Importantly, biopsy specimens from patients who developed progressive disease after first-line amivantamab–lazertinib also showed increased HER2 protein levels, and in vitro experiments confirmed that *HER2* overexpression mediates resistance. Notably, a patient who progressed on first-line therapy with documented *HER2* overexpression and amplification subsequently achieved stable disease when treated with a *HER2*-targeted TKI. These transcriptomic and genomic findings, considered together, suggest that increased *HER2* signalling plays a role in amivantamab–lazertinib resistance. This evidence suggests the possibility of effective therapeutic strategies that target the *HER2* pathway for patients who experience disease progression following this combination therapy [17].

Much of our existing understanding regarding how resistance to amivantamab develops is derived from clinical observations of individuals with NSCLC harboring *EGFR* exon 20 insertion (ex20ins) mutations. In this specific patient group, the bispecific antibody serves as a primary therapeutic option following the failure of platinum-based chemotherapy. A recent prospective analysis utilized extensive ctDNA genomic monitoring to evaluate ten patients in this category, comparing molecular profiles from baseline samples with those obtained at the time of disease progression [18]. Within this cohort, 80% of participants (eight patients) exhibited a baseline *EGFR* ex20ins variant allele frequency (VAF) of 1% or higher, whereas the remaining 20% (two patients) had a VAF below this threshold. Furthermore, *EGFR* amplification was identified in half of the study population. For those in the high-VAF category, progression was marked by a wide spectrum of molecular changes, specifically the amplification of *MET*, *MYC*, *PIK3CA*, and *KRAS*, alongside *EP300* deletions. Genomic modifications impacting the cell cycle—including *RB1*, *CCNE1*, *CDK4/6*, and *CCND1/2/3*—were also observed. While two individuals in this group had no detectable genetic drivers of resistance at the time of progression, those in the low-VAF group (< 1%) showed even fewer simultaneous alterations: one patient developed a *KRAS* G13C mutation, while the other showed no identifiable resistance markers. Collectively, the patterns of resistance were highly varied and primarily characterized by the activation of bypass signaling routes, often involving multiple simultaneous molecular changes. In an effort to identify how resistance to amivantamab develops, researchers performed an extensive evaluation of 12 patients with *EGFR* ex20ins NSCLC, comparing genomic data from baseline against samples taken at the time of disease progression. The analysis showed that 58.3% of these patients (seven individuals) acquired at least one new candidate resistance mutation that was absent before treatment. Among these, *EP300* deletions were the most common finding, identified in 25.0% of the group (*n* = 3). When examining specific biological routes of escape, several pathways were frequently implicated: genomic changes in RTK/RAS/PI3K/AKT signaling, the p53 pathway, and cell cycle regulation were each observed in 41.6% of patients (five individuals each), while alterations in Hippo/NOTCH signaling were found in 25.0% of cases (three individuals). In-depth evaluation of individual cases uncovered a wide variety of escape routes following amivantamab therapy. In one instance, a patient acquired a secondary *EGFR P281L* mutation simultaneously with *ARID1A Y560H* and CHEK2 R95* mutations. Isolated resistance markers were found in 16.7% of the cohort (two patients), with one individual carrying a *PIK3CA H1074R* mutation and the other an *RB1 M851I* mutation. Remarkably, one-third of the patients (33.3%) demonstrated highly complex resistance profiles characterized by multiple simultaneous alterations across different bypass pathways. Within this same percentage of the group, genomic changes in bypass signaling genes appeared alongside modifications in the RTK/RAS/PI3K/AKT axis. For example, one patient presented a *TSC1* deletion in combination with *CDKN2A, EP300, NF2*, and *CHEK2* deletions. Another case involved *ERBB2 Q1027H* with amplifications of *RICTOR* and *PIK3CA*, occurring alongside *MDM2* and RB1 K4* amplifications. Two other individuals showed various RTK/RAS/PI3K/AKT signaling modifications coupled with several bypass pathway shifts.

These results highlight the remarkable heterogeneity of acquired resistance mechanisms to amivantamab. Some patients develop single, well-defined resistance alterations, whereas others acquire multiple concurrent changes affecting both canonical RTK/RAS/PI3K/AKT signaling and bypass pathways. The co-occurrence of alterations across multiple pathways, as observed in several patients, underscores the complexity of resistance and suggests that monotherapy targeting a single node may be insufficient in some cases. These findings also point to the potential need for combination strategies or sequential therapies guided by serial molecular profiling to overcome or delay resistance.

## Strategies of treatment at the establishment of resistance to *EGFR* TKIs

Although significant progress has been made in the frontline management of *EGFR*-driven NSCLC, the development of acquired resistance continues to be an inescapable clinical hurdle. To address this, several innovative therapeutic strategies are currently being explored, focusing on the inhibition of tertiary mutations, the application of bispecific antibodies, and the utilization of antibody-drug conjugates (ADCs). Current evidence regarding these next-generation agents primarily stems from clinical trials assessing their performance following the failure of first-line *EGFR* TKIs. Among these, bispecific antibodies designed to concurrently block two distinct pathways associated with drug resistance have shown considerable promise. For instance, the MARIPOSA-2 study evaluated amivantamab in combination with chemotherapy for patients in the second-line setting. This combination therapy achieved a superior objective response rate (ORR) of 64%, compared to 36% with chemotherapy alone, and extended PFS to 6.3 months from 4.2 months in the control group [19]. Furthermore, the HARMONi-A trial [20] demonstrated that simultaneously blocking PD-1 and vascular endothelial growth factor receptor (VEGFR) with ivonescimab significantly enhanced clinical outcomes when added to chemotherapy in the second-line setting. This combination therapy achieved a median PFS of 6.8 months compared to 4.4 months for chemotherapy alone (HR 0.52, *P* < 0.0001), while also improving the ORR to 50.6% from 35.4%. Additionally, a favourable trend in median survival was observed, with the ivonescimab arm reaching 16.8 months versus 14 months in the control group.

Because *MET* amplification or overexpression represents a primary driver of resistance to third-generation *EGFR* TKIs, researchers are actively evaluating therapeutic strategies that combine selective *MET* inhibitors with the continued use of *EGFR* TKIs [21–23]. Clinical benefit has been observed in early-phase investigations involving tepotinib within the INSIGHT 2 study [24] and savolitinib during the phase Ib TATTON trial. The phase II SAVANNAH study further validated the advantages of this therapeutic pairing specifically for individuals exhibiting elevated *MET* expression. Looking forward, the phase III SAFFRON trial is designed to establish the clinical value of combining savolitinib with osimertinib compared to standard chemotherapy as a subsequent intervention for those who have progressed on osimertinib (ClinicalTrials.gov identifier: NCT05261399).

**Figure 1:**
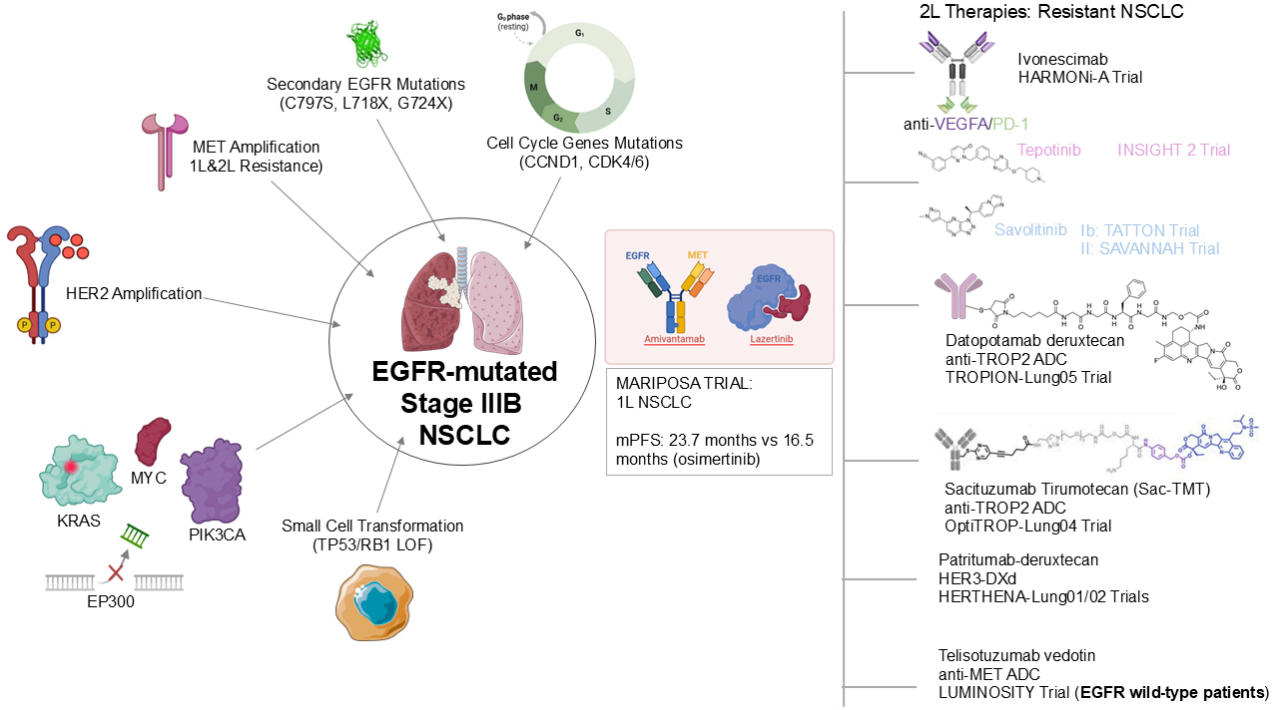
**Mechanisms of resistance in *EGFR*-mutated NSCLC and therapeutic strategies.** The central image depicts an *EGFR*-mutated NSCLC cell (stage IIIB), surrounded by various mechanisms of acquired resistance, including secondary *EGFR* mutations (C797S, L718X, G724X), *MET* amplification (1L & 2L resistance), *HER2* amplification, cell cycle gene mutations (*CCND1*, *CDK4/6*), *KRAS* and *PIK3CA* mutations, *EP300* deletions, and small cell transformation (TP53/RB1 LOF). The MARIPOSA trial, combining amivantamab and lazertinib for 1L NSCLC, is highlighted with key efficacy data [mPFS: 23.7 months vs. 16.5 months (osimertinib)]. On the right, 2L+ therapeutic strategies for resistant NSCLC are listed, featuring agents like ivonescimab (anti-VEGFA/PD-1) in the HARMONi-A trial, tepotinib (INSIGHT 2 trial), savolitinib (Ib: TATTON trial; II: SAVANNAH trial), datopotamab deruxtecan (anti-TROP2 ADC) in TROPION-Lung05 trial, sacituzumab tirumotecan (Sac-TMT, anti-TROP2 ADC) in OptiTROP-Lung04 trial, patritumab deruxtecan (HER3-DXd) in HERTHENA-Lung01/02 trials, and telisotuzumab vedotin (anti-MET ADC) in the LUMINOSITY trial (for *EGFR* wild-type patients). The graphical scheme was produced by the authors. Some elements in this figure were drawn using pictures from Servier Medical Art by Servier, which is licensed under a Creative Commons Attribution 3.0 Unported License (https://creativecommons.org/licenses/by/3.0/).

A third class of agents involved in clinical investigation in this setting is represented by ADCs, which provide targeted delivery of cytotoxic agents to tumours. In particular, *EGFR*-mutant adenocarcinoma tends to have higher and more uniform *TROP2* expression, with the *TROP2* expression remaining across resistance clones serving as a stable target and ensuring a better TROP2 ADC target engagement. Datopotamab-deruxtecan, a TROP2-directed ADC conjugated to a topoisomerase I inhibitor deruxtecan payload, achieved an ORR of 43.6% with a mPFS of 5.8 months and median OS of 18.3 months, in *EGFR*-mutant NSCLC after *EGFR* TKI and chemotherapy, in TROPION-Lung05 [25]. The pivotal phase III OptiTROP-Lung04 trial, recently showcased at the ESMO Congress 2025 [26], assessed the safety and clinical impact of Sac-TMT, an advanced ADC targeting *TROP2*. This agent utilizes a specialized cleavable linker to deliver a belotecan-derived topoisomerase I inhibitor, a design intended to maximize tumor-specific lethality while reducing off-target systemic effects. When compared to standard platinum-based chemotherapy in *EGFR*-mutated NSCLC patients who failed previous TKI therapy, Sac-TMT achieved statistically superior results. Specifically, it extended median PFS to 8.3 months from 4.3 months (HR 0.49; *P* < 0.0001) and demonstrated a significant OS advantage, with the median survival not yet reached in the Sac-TMT group compared to 17.4 months in the chemotherapy arm (HR 0.60; *P* = 0.001). These outcomes suggest that Sac-TMT may become a foundational treatment for this resistant patient subgroup [27]. In a similar way, the HERTHENA-Lung01 trial highlighted the potential of patritumab-deruxtecan (HER3-DXd), which produced lasting and significant responses in individuals with *EGFR*-mutated NSCLC who had previously undergone both TKI and chemotherapy. Nevertheless, the subsequent phase III HERTHENA-Lung02 study did not confirm an OS benefit for *HER3*-DXd when compared against platinum-based chemotherapy in patients progressing after third-generation TKI therapy [28]. Lastly, the LUMINOSITY trial explored the potential of telisotuzumab vedotin, an ADC designed to target *MET*, specifically for patients with *EGFR*-mutated NSCLC who had already failed *EGFR* TKI therapy. The investigation revealed that in cases characterized by *MET* overexpression, the agent achieved an ORR of 28.6% and a median PFS of 5.7 months [29] (Figure 1).

Recently, fourth-generation *EGFR* TKI targeting *C797S* mutations, which drive resistance in 10%–15% of patients pretreated with osimertinib, are in development in order to selectively bind to this mutation.

In a phase I clinical evaluation, the agent BDTX-1535 demonstrated clinical activity, achieving partial responses in five out of 15 participants and maintaining stable disease in an additional nine patients [30]. Several other novel compounds are currently being studied, such as JINA02 (NCT05394831), BAY2927088 (NCT05099172), and BPI-361175 (NCT05393466), as part of ongoing efforts to address treatment resistance. The clinical development of frontline combination strategies aims to proactively delay resistance; the design, efficacy, and safety profiles of these pivotal trials, along with subsequent line interventions, are summarized in Table 1.

**Table 1:** Summary of pivotal and emerging clinical trials for the management of *EGFR*-mutant NSCLC.

**Trial (Phase)**	**Study design & regimen**	**Major outcomes (mPFS/mOS)**	**Acquired resistance mechanisms**	**Key toxicities and considerations**
FLAURA2 (III) [12]	1L: osimertinib + platinum-pemetrexed vs. osimertinib	PFS: 25.5 vs. 16.7 months; OS: 47.5 vs. 37.6 months		Predominantly C797S and MET alterations; chemotherapy delays but does not change the molecular profile.
MARIPOSA (III) [15, 16]	1L: amivantamab + lazertinib vs. osimertinib	PFS: 23.7 vs. 16.6 months; OS: NR vs. 36.7 months	Significantly reduced *MET* amp (4.4% vs. 13.6%) and secondary *EGFR* mutations (0.9% vs. 7.9%).	Infusion-related reactions (IRRs) and dermatological events; utilizes “dual” receptor inhibition.
MARIPOSA-2 (III)	2L (post-osi): amivantamab + chemo ± lazertinib vs. chemo	PFS: 6.3 vs. 4.2 months	Heterogeneous bypass pathway activation.	Managed IRRs; established a new second-line standard.
HARMONi-A (III) [20]	2L: ivonescimab (PD-1/VEGF) + chemo vs. chemo	PFS: 6.8 vs. 4.4 months; OS: 16.8 vs. 14.0 months (trend) + 1	Bypass signaling and RTK/RAS/PI3K pathway modifications.	VEGF-related adverse events (hypertension, proteinuria).
OptiTROP-Lung04 (III) [26]	2L: Sac-TMT (TROP2-ADC) vs. platinum-based chemo	PFS: 8.3 vs. 4.3 months; OS: NR vs. 17.4 months	TROP2 expression remains stable across resistance clones.	Potential for tumor-selective cytotoxicity with reduced systemic toxicity.
TROPION-Lung05 (II) [25]	2L: datopotamab deruxtecan (TROP2-ADC)	PFS: 5.8 months; OS: 18.3 months	Topoisomerase I inhibitor payload engagement.	Stomatitis and ocular toxicities are common to the ADC class.
INSIGHT 2 (II) [24]	2L (*MET*+): tepotinib + osimertinib	ORR: 54.8% (in *MET*-amplified)	Targets acquired *MET*-mediated bypass resistance.	Peripheral edema is a known class effect of MET inhibitors.

This table provides a comparative overview of frontline (1L) and second-line (2L) regimens, highlighting efficacy outcomes (mPFS, mOS), mechanisms of acquired resistance suppressed or observed, and primary safety considerations to guide clinical stratification. ADC: antibody-drug conjugate; *EGFR*: epidermal growth factor receptor; IRR: infusion-related reaction; *MET*: MET proto-oncogene; mOS: median overall survival; mPFS: median progression-free survival; NR: not reached; NSCLC: non-small cell lung cancer; ORR: objective response rate; PD-1: programmed cell death protein 1; RTK: receptor tyrosine kinase; Sac-TMT: sacituzumab tirumotecan; *TROP2*: trophoblast cell-surface antigen 2; *VEGF*: vascular endothelial growth factor.

## Conclusions

Our deepening comprehension of *EGFR*-mutated NSCLC has fundamentally transformed clinical management, ushering in a sophisticated era of precision oncology. While the expanding range of therapeutic options offers significant hope, it also increases the complexity of clinical decision-making, presenting clinicians with both opportunities and novel obstacles. The upcoming phase of research must focus on neutralizing resistance mechanisms while personalizing care to meet individual patient objectives, ensuring a meticulous balance between treatment effectiveness, manageable toxicity, and preserved quality of life. Also, updated knowledge on resistance mechanisms should be investigated in parallel with clinical data to speed up the next steps of clinical research and improve treatment options faster for patients.

## Abbreviations

ADCs: antibody-drug conjugates
ctDNA: circulating tumor DNA
DTP: drug-tolerant persister
ex20ins: exon 20 insertion
NSCLC: non-small cell lung cancer
ORR: objective response rate
OS: overall survival
PDX: patient-derived xenograft
PFS: progression-free survival
PI3K: phosphatidylinositol 3-kinase
SCLC: small-cell lung cancer
TKIs: tyrosine kinase inhibitors
VAF: variant allele frequency

## Declarations

### Copyright

© The Author(s) 2026.

## References

[1] Morgillo F, Della Corte CM, Fasano M, Ciardiello F (2016). Mechanisms of resistance to EGFR-targeted drugs: lung cancer. ESMO Open.

[2] Chmielecki J, Gray JE, Cheng Y, Ohe Y, Imamura F, Cho BC (2023). Candidate mechanisms of acquired resistance to first-line osimertinib in EGFR-mutated advanced non-small cell lung cancer. Nat Commun.

[3] Zhang Q, Zhang XC, Yang JJ, Yang ZF, Bai Y, Su J (2018). EGFR L792H and G796R: Two Novel Mutations Mediating Resistance to the Third-Generation EGFR Tyrosine Kinase Inhibitor Osimertinib. J Thorac Oncol.

[4] Peng D, Liang P, Zhong C, Xu P, He Y, Luo Y (2022). Effect of EGFR amplification on the prognosis of EGFR-mutated advanced non-small-cell lung cancer patients: a prospective observational study. BMC Cancer.

[5] Xu CW, Lei L, Wang WX, Lin L, Zhu YC, Wang H (2020). Molecular Characteristics and Clinical Outcomes of EGFR Exon 19 C-Helix Deletion in Non-Small Cell Lung Cancer and Response to EGFR TKIs. Transl Oncol.

[6] Eberlein CA, Stetson D, Markovets AA, Al-Kadhimi KJ, Lai Z, Fisher PR (2015). Acquired Resistance to the Mutant-Selective EGFR Inhibitor AZD9291 Is Associated with Increased Dependence on RAS Signaling in Preclinical Models. Cancer Res.

[7] Papadimitrakopoulou VA, Wu YL, Han JY, Ahn MJ, Ramalingam SS, John T (2018). Analysis of resistance mechanisms to osimertinib in patients with EGFR T790M advanced NSCLC from the AURA3 study. Ann Oncol.

[8] Minari R, Bordi P, Del Re M, Facchinetti F, Mazzoni F, Barbieri F (2018). Primary resistance to osimertinib due to SCLC transformation: Issue of T790M determination on liquid re-biopsy. Lung Cancer.

[9] Niederst MJ, Sequist LV, Poirier JT, Mermel CH, Lockerman EL, Garcia AR (2015). RB loss in resistant EGFR mutant lung adenocarcinomas that transform to small-cell lung cancer. Nat Commun.

[10] Planchard D, Jänne PA, Cheng Y, Yang JC, Yanagitani N, Kim SW, FLAURA2 Investigators (2023). Osimertinib with or without Chemotherapy in EGFR-Mutated Advanced NSCLC. N Engl J Med.

[11] Jänne PA, Planchard D, Kobayashi K, Yang JC, Liu Y, Valdiviezo N, FLAURA2 Investigators (2026). Survival with Osimertinib plus Chemotherapy in EGFR-Mutated Advanced NSCLC. N Engl J Med.

[12] Yang JC, Robichaux J, Planchard D, Kobayashi K, Lee CK, Sugawara S (2024). MA12. 03 FLAURA2: resistance, and impact of baseline TP53 alterations in patients treated with 1L Osimertinib ± platinum-pemetrexed. J Thorac Oncol.

[13] Lee CK, Robichaux JP, Jänne PA, Kim SW, Kim TM, Kobayashi K (2023). 514MO Acquired mechanisms of resistance to first-line (1L) osimertinib with or without platinum-based chemotherapy (CT) in EGFR-mutated (EGFRm) advanced NSCLC: preliminary data from FLAURA2. Ann Oncol.

[14] Cho BC, Sethi S, Felip E (2024). Amivantamab plus lazertinib in previously untreated EGFR-mutated advanced NSCLC. N Engl J Med.

[15] Wiesweg M, Gadgeel S, Cho B, Lu S, Felip E, Hayashi H (2025). Amivantamab Plus Lazertinib vs Osimertinib in First-line EGFR-mutant Advanced NSCLC: Longer Follow-up of the MARIPOSA Study. Pneumologie.

[16] Besse B, Lee SH, Lu S, Stroyakovskiy D, Yazici O, Cid JR (2024). LBA55 Mechanisms of acquired resistance to first-line amivantamab plus lazertinib versus osimertinib in patients with EGFR-mutant advanced non-small cell lung cancer: an early analysis from the phase III MARIPOSA study. Ann Oncol.

[17] Lim SM, Shim JSG, Lee YW, Cho BC, Yun M (2025). 111P HER2 overexpression drives drug-tolerant persisters to first-line amivantamab and lazertinib in EGFR-mutant non-small cell lung cancer. ESMO Open.

[18] Park GH, Park S, Kim H, Jung HA, Sun JM, Ahn JS (2025). Prospective investigation of biomarker and resistance mechanism using longitudinal cell-free NGS in non-small cell lung cancer with EGFR exon 20 insertion treated with amivantamab. Eur J Cancer.

[19] Passaro A, Wang J, Wang Y, Lee SH, Melosky B, Shih JY, MARIPOSA-2 Investigators (2024). Amivantamab plus chemotherapy with and without lazertinib in EGFR-mutant advanced NSCLC after disease progression on osimertinib: primary results from the phase III MARIPOSA-2 study. Ann Oncol.

[20] Fang W, Zhao Y, Luo Y, Yang R, Huang Y, He Z, HARMONi-A Study Investigators (2024). Ivonescimab Plus Chemotherapy in Non-Small Cell Lung Cancer With EGFR Variant: A Randomized Clinical Trial. JAMA.

[21] Hartmaier RJ, Markovets AA, Ahn MJ, Sequist LV, Han JY, Cho BC (2023). Osimertinib + Savolitinib to Overcome Acquired MET-Mediated Resistance in Epidermal Growth Factor Receptor-Mutated, MET-Amplified Non-Small Cell Lung Cancer: TATTON. Cancer Discov.

[22] Yu HA, Ambrose H, Baik C, Cho BC, Cocco E, Goldberg SB (2021). 1239P ORCHARD osimertinib+ savolitinib interim analysis: A biomarker-directed phase II platform study in patients (pts) with advanced non-small cell lung cancer (NSCLC) whose disease has progressed on first-line (1L) osimertinib. Ann Oncol.

[23] Ahn MJ, De Marinis F, Bonanno L, Cho BC, Kim TM, Cheng S (2022). EP08. 02-140 MET biomarker-based preliminary efficacy analysis in SAVANNAH: savolitinib+ osimertinib in EGFRm NSCLC post-osimertinib. J Thorac Oncol.

[24] Wu Y, Guarneri V, Voon PJ, Lim BK, Yang JJ, Wislez M (2024). Tepotinib plus osimertinib in patients with EGFR-mutated non-small-cell lung cancer with MET amplification following progression on first-line osimertinib (INSIGHT 2): a multicentre, open-label, phase 2 trial. Lancet Oncol.

[25] Sands J, Ahn MJ, Lisberg A, Cho BC, Blumenschein G Jr, Shum E (2025). Datopotamab Deruxtecan in Advanced or Metastatic Non-Small Cell Lung Cancer With Actionable Genomic Alterations: Results From the Phase II TROPION-Lung05 Study. J Clin Oncol.

[26] Zhang L, Fang WF, Wu L, Meng X, Yao Y, Zuo W (2025). LBA5 Sacituzumab tirumotecan (sac-TMT) vs platinum-based chemotherapy in EGFR-mutated (EGFRm) non-small cell lung cancer (NSCLC) following progression on EGFR-TKIs: results from the randomized, multi-center phase III OptiTROP-Lung04 study. Ann Oncol.

[27] Yu HA, Goto Y, Hayashi H, Felip E, Chih-Hsin Yang J, Reck M (2023). HERTHENA-Lung01, a Phase II Trial of Patritumab Deruxtecan (HER3-DXd) in Epidermal Growth Factor Receptor-Mutated Non-Small-Cell Lung Cancer After Epidermal Growth Factor Receptor Tyrosine Kinase Inhibitor Therapy and Platinum-Based Chemotherapy. J Clin Oncol.

[28] Mok TS, Yu HA, Lim SM, Okamoto I, Perol M, Novello S (2025). Patritumab deruxtecan (HER3-DXd) in resistant *EGFR*-mutated (*EGFR*m) advanced non-small cell lung cancer (NSCLC) after a third-generation EGFR TKI: The phase 3 HERTHENA-Lung02 study. J Clin Oncol.

[29] Camidge DR, Bar J, Horinouchi H, Goldman J, Moiseenko F, Filippova E (2024). Telisotuzumab Vedotin Monotherapy in Patients With Previously Treated c-Met Protein-Overexpressing Advanced Nonsquamous EGFR-Wildtype Non-Small Cell Lung Cancer in the Phase II LUMINOSITY Trial. J Clin Oncol.

[30] Johnson ML, Henry JT, Spira AI, Battiste J, Alnahhas I, Ahluwalia MS (2023). A phase 1 study to assess BDTX-1535, an oral EGFR inhibitor, in patients with glioblastoma or non–small-cell lung cancer. J Clin Oncol.

